# Nano-encapsulated *Escherichia coli* Divisome Anchor ZipA, and in Complex with FtsZ

**DOI:** 10.1038/s41598-019-54999-x

**Published:** 2019-12-10

**Authors:** Sarah C. Lee, Richard Collins, Yu-pin Lin, Mohammed Jamshad, Claire Broughton, Sarah A. Harris, Benjamin S Hanson, Cecilia Tognoloni, Rosemary A. Parslow, Ann E. Terry, Alison Rodger, Corinne J. Smith, Karen J. Edler, Robert Ford, David I. Roper, Timothy R. Dafforn

**Affiliations:** 1 School of Biosciences, University of Birmingham, Edgbaston, Birmingham B15 2TT UK; 2 Faculty of Life Sciences, A4032 Michael Smith Building, Oxford Road, Manchester, M13 9PT UK; 3 School of Life Sciences, University of Warwick, Gibbet Hill Road, Coventry, CV4 7AL UK; 4 School of Physics and Astronomy and Astbury Centre for Structural and Molecular Biology, University of Leeds, Leeds, UK; 5 Department of Chemistry, University of Bath, Claverton Down, Bath, BA2 7AY UK; 6 MAX IV Laboratory Lund University, P.O. Box 118, SE-221 00, Lund, Sweden; 7 Department of Molecular Sciences, Macquarie University, Macquarie, NSW 2109 Australia

**Keywords:** Cryoelectron microscopy, Membrane structure and assembly, Cellular microbiology

## Abstract

The *E*. *coli* membrane protein ZipA, binds to the tubulin homologue FtsZ, in the early stage of cell division. We isolated ZipA in a Styrene Maleic Acid lipid particle (SMALP) preserving its position and integrity with native *E*. *coli* membrane lipids. Direct binding of ZipA to FtsZ is demonstrated, including FtsZ fibre bundles decorated with ZipA. Using Cryo-Electron Microscopy, small-angle X-ray and neutron scattering, we determine the encapsulated-ZipA structure in isolation, and in complex with FtsZ to a resolution of 1.6 nm. Three regions can be identified from the structure which correspond to, SMALP encapsulated membrane and ZipA transmembrane helix, a separate short compact tether, and ZipA globular head which binds FtsZ. The complex extends 12 nm from the membrane in a compact structure, supported by mesoscale modelling techniques, measuring the movement and stiffness of the regions within ZipA provides molecular scale analysis and visualisation of the early divisome.

## Introduction

The assembly of the mega-protein complex^1^ known as the divisome, required to initiate and complete bacterial cell division, remains of fundamental interest for biochemists and microbiologists alike and is a key to designing molecular targets to control cell proliferation. The temporal and spatial complexity of this process requires exquisite control of a number of protein complexes and their interaction with the membrane in order to complete septa formation and fission. Central to this process is the construction and tethering of the Z-ring formed of polymeric FtsZ to the cell membrane at the cell midline^2–4^. Current data on the formation of the divisome derives mainly from genetic, biochemical and microscopy studies and indicates that it forms through a sequential recruitment of proteins^5–7^. Recently, landmark studies have provided a connection between the “treadmilling” polymerisation of FtsZ and septal peptidoglycan biosynthesis providing a direct linkage between cell division and cell wall biosynthesis^8,9^. With respect to *E*. *coli*, this begins with the formation of the Z-ring in concert with recruitment of the membrane tethers ZipA followed by FtsA^10^. These proteins are joined by the subcomplex of FtsE:FtsX, which structurally resembles an ABC transporter^11–13^. The FtsE:FtsX sub-complex is responsible for localisation of extracellular peptidoglycan hydrolases required for separation of daughter cells. This stage is followed by recruitment of FtsK, which is involved in chromosome separation^14^, and is able to recruit a heterotrimeric complex containing FtsQ:FtsB:FtsL^15^. Completion of this sub-complex assembly enables the addition of FtsW^16^, FtsI (PBP3)^17^ and PBP1b^18^, required for new cell wall peptidoglycan biosynthesis and additional peptidoglycan hydrolases, AmiC and EnvC^19,20^. One of the last proteins to be recruited to the divisome is the essential protein FtsN^21,22^ which is thought to interact with various parts of the sub-complexes (ZipA:FtsZ:FtsA, FtsQ:FtsL:FtsB) as well as divisome associated penicillin binding proteins. Thus, FtsN potentially plays a role as both recruiter and regulator of various aspects of the divisome, providing linkage to other important proteins associated with cell division and repair assembly.

Despite this clear definition of recruitment steps, there remains a significant gap in our knowledge and understanding of this process at a molecular level. This can be attributed to the significant challenges concomitant with structural and biochemical studies of the many protein components that are embedded in the inner membrane.

**Figure 1:**
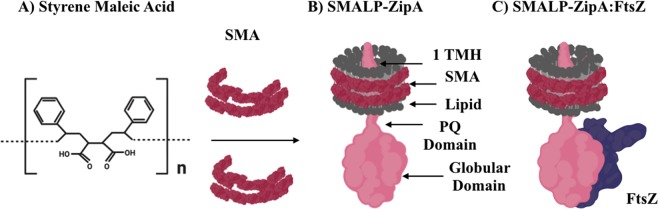
The schematic representation of SMALP-ZipA and SMALP-ZipA:FtsZ. (**A**) The chemical structure of SMA co-polymer which has a ratio of 2:1 styrene to maleic acid (adapted from Lee *et al*.)^39^. (**B**) Schematic representation of SMALP-ZipA (created using BioRender.com) 1TMH is encapsulated in native lipid (grey) from the *E*. *coli* membrane, and surrounded by an SMA disc (red). ZipA (pink) shows the first 6 amino acids exposed on the periplasmic side of the membrane, aa 7–28 form a 1TMH, 29–85 aa lead into the cytoplasmic region of the cell, and are connected by a flexible linker, the PQ domain, to the globular domain (GD) consisting of 186–328 aa. (**C**) Schematic representation of the SMALP-ZipA:FtsZ complex.

In 2009^23^ we showed with others^24^ that a low cost amphipathic polymer, styrene maleic acid co-polymer (SMA) (Fig. 1A) can be used to excise 10 nm diameter nano-discs from phospholipid bilayers. We showed that these nano-discs contained a stabilised lipid bilayer which can support functional membranes proteins (MP’s) of a wide range and types. We also showed that SMALP’s provided a material amenable to study using electron microscopy yielding medium resolution protein structures^25^. We now deploy this methodology in the study of the early bacterial divisome assembly.

ZipA is a membrane anchored 36.4 kDa protein found in gammaproteobacteria, including *E. coli* and consists of 328 amino acid (aa) residues, and along with FtsA is required for FtsZ tethering to the membrane in *E*. *coli*^26,27^. The protein can be defined from the N-terminal; The first 6 aa are exposed to the periplasmic side of the membrane, aa 7–28 form a single transmembrane helix (TMH) which serves to tether the protein within the inner membrane. A set of charged aa residues, between 29–85 link a region of 100 aa, residues 86–185^28^. This region is predicted to be intrinsically unstructured, rich in proline and glutamine and in the ZipA sequence referred to as the “PQ” domain^29^. Residues 186–328 form a globular domain (GD) or FtsZ-binding domain (FZB), which binds the C-terminal of the cytoskeletal protein FtsZ^4^. The ZipA GD may have a number of functions. Firstly, to bind to the C-terminus of FtsZ *via* a conserved carboxy-terminal peptide (CCTP)^4,30,31^ providing a membrane tether for the FtsZ ring. Secondly, the formation of this complex alters the self-association within the ring inducing fibre bundling^32^. Thirdly, ZipA has been shown to protect FtsZ fibres from digestion by ClpXP-directed degradation, enhancing their stability^33^. Finally, it has been proposed that ZipA stabilizes FtsZ which then forms membrane-attached spiral-like structures in the bacterial cytoplasm^33^.

Studies have shown that ZipA is able to respond to changes in membrane fluidity by altering the conformation of the PQ region, allowing it to adopt an extended, brush-like, conformation in response to increased membrane packing^34^. It is hypothesised that this conformational change could be triggered during cell division, allowing ZipA to act as an entropic seeker^34^, enhancing its efficiency in capturing FtsZ fibres. In addition, the added flexibility of attachment^35^ has the potential to facilitate FtsZ fibre packing.

In more recent studies, attempts have been made to reconstitute the division machinery into vesicle systems in order to examine the division process itself^21^. A modified version of ZipA where the transmembrane domain was replaced by a membrane surface associated motif, showed accumulation in regions of structural constriction in the vesicle. Most recently a ZipA:FtsZ reconstitution system led to the observation of vesicle shrinkage and membrane ruffling^21^. Hernández-Rocamora *et al*.^36^ have attempted to resolve this issue by using a membrane scaffold protein (MSP) nano-disc methodology^37^ that uses peptides to extract MP’s into nanoscale particles that contain a membrane bilayer^38^. This system seemed unsuited for structural studies, since although it produced a negative stain TEM reconstructions from ZipA nanodiscs, the structure did not differ significantly from that of empty nanodiscs. The study did however, confirm that when encapsulated in the nanodisc the ZipA maintained FtsZ binding activity although no FtsZ bundling activity was observed.

In this work we show how structural and biochemical insights into the early stage of divisome formation can be enabled by the deployment of a new technique for producing MP’s using SMALPs. Specifically, we use the SMA co-polymer to extract and purify the divisomal anchor protein ZipA into stable nano-discs encircled by SMA polymer. Figure 1B provides a schematic representation of the proposed known architecture of ZipA represented in a SMALP (SMALP-ZipA), where ZipA is encapsulated in *E*. *coli* native lipids (grey) encircled by SMA forming a disc (red). In addition, we have further heterologously expressed and purified FtsZ, which is amenable to polymerisation with Guanosine-5′-triphosphate (GTP).

We demonstrate the extraction of full length ZipA from *E*. *coli* membranes using the SMALP methodology. SMALP-ZipA can be purified by affinity chromatography and size exclusion chromatography (SEC). SMALP-ZipA binds to FtsZ and forms a stable complex. Figure 1C provides a schematic representation of SMALP-ZipA bound to FtsZ.

The addition of SMALP-ZipA to FtsZ in presence of GTP induces bundling of FtsZ into fibres. Negative stain transmission electron microscopy analysis (TEM) of SMALP-ZipA shows it decorates FtsZ fibres at regular intervals, and increases FtsZ fibre formation. Increasing the concentration of SMALP-ZipA increased FtsZ fibre formation which progressed to fibre bundling. We have further elucidated the structure of SMALP-ZipA and SMALP-ZipA:FtsZ representing formation of a proto-ring as an early divisome event, using three orthologous techniques, Small Angle X-ray Scattering (SAXS), Small Angle Neutron Scattering (SANS) and Cryo-EM. These orthologous methods show a high degree of complementary fit and reveal that the SMALP-ZipA:FtsZ complex is short and compact in contrast to previous observations. Fluctuating Finite Element Analysis (FFEA) modelling of the complex suggests that some flexibility exists in the PQ domain allowing limited entropic seeking but that this flexibility is removed upon binding of FtsZ.

## Results

### ZipA can be encapsulated into SMALPs and purified from *E*. *coli*

**Figure 2:**
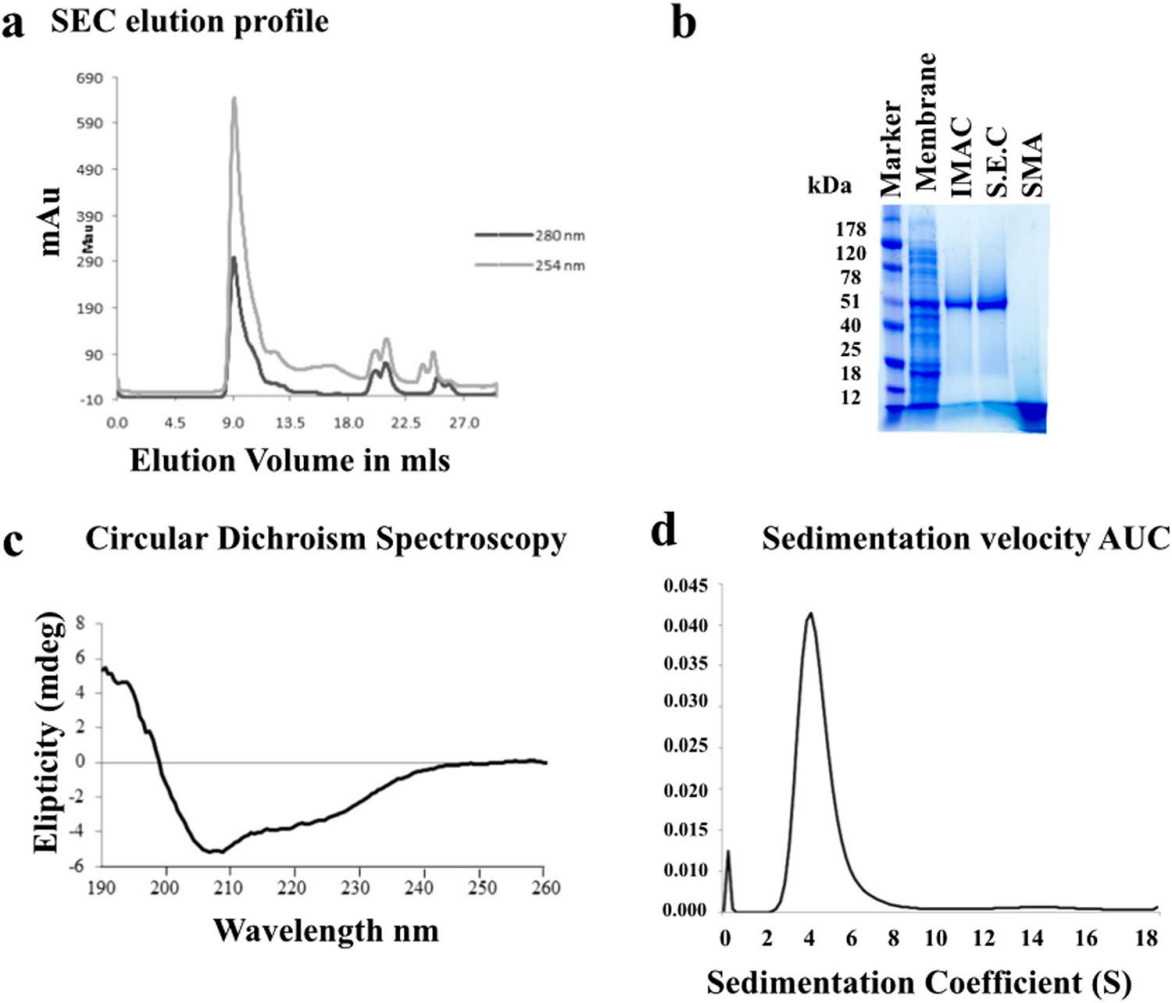
Purification and characterization of SMALP-ZipA. (**a**) Elution profile of SEC chromatography using Superdex 200 Increase 10/300 column. (**b**) SDS-PAGE stained with Coomassie blue, left to right, protein standards marker (Marker), crude membrane fraction containing over-expressed ZipA (Membrane), SMALP-ZipA protein from the IMAC step (IMAC), SMALP-ZipA from the SEC step (SEC), and 2.5% wt/vol SMA alone (SMA). (**c**) CD data shows purified SMALP-ZipA to consist of alpha helices, beta sheets and unstructured regions, consistent with the predicted structure of ZipA. (**d**) Sedimentation velocity AUC (Figure adapted from Lee *et al*. Nature Protocols 2016).

ZipA was successfully overexpressed in *E*. *coli* and directly extracted from membranes with the addition of 2.5% SMA wt/vol following the protocol of Lee *et al*.^39^. Purification was carried out using Ni-affinity chromatography, followed by SEC. 250 µl of sample was loaded at a flow rate of 0.5 ml.min^–1^. The protein elution was monitored by absorbance at 280 nm and 254 nm, enabling the detection of both proteins in SMALP (280 nm) and free SMA (254 nm). The trace shows that SMALP-ZipA is effectively separated from free SMA. (Fig. 2a), samples were analysed by sodium dodecyl sulfate polyacrylamide gel electrophoresis (SDS-PAGE). The final purity of the SMALP-ZipA sample was judged from SDS-PAGE to be in excess of 95%. The identity of the protein was confirmed as ZipA by peptide mass spectrometry of the major band from SDS-PAGE^40,41^. The proteomics data yielded 100% sequence coverage of the ZipA peptide. It was noted that the protein migrates on SDS-PAGE with an apparent mass of 52 kDa (Fig. 2b lane IMAC and SEC). The discrepancy between this and the actual weight of our construct (39.5 kDa including tags)^32^ is not unexpected given that MP’s commonly migrate anomalously on SDS-PAGE. The SMA polymer can be identified as a diffuse band at approximately 8 kDa, and is also present in the IMAC and SEC lanes, where it has separated from the SMALP-ZipA during SDS-PAGE.

A circular dichroism (CD) spectrum was measured for SMALP-ZipA to establish that the sample contained folded protein (Fig. 2c). We note from the CD spectrum obtained that there is significant negative intensity at 222 nm which corresponds to alpha helix content; the 195 nm region is lower than usual meaning there must be a negative contribution from either unfolded or polyproline sheet of beta-II; the 218 nm region is less of a structured dip than for a pure alpha helix so we deduce the presence of beta sheet which is consistent with the predicted structure of ZipA. Sedimentation velocity analytical ultracentrifugation (SvAUC) studies of SEC purified SMALP-ZipA were analysed with SEDFIT^42,43^ using the c(S) and c(M) routines revealed that SMALP-ZipA is present as a single species with a sedimentation coefficient of 4.0 S. and a molecular mass of ~70 kDa for the SMALP-ZipA. This suggests that the SMA and lipids present in the SMALP contribute 30–35 giving a total mass of 100 kDa. It suggests purified SMALP-ZipA maintained its native secondary structure after solubilisation and purification (Fig. 2d).

### Functional testing of SMALP-ZipA

**Figure 3:**
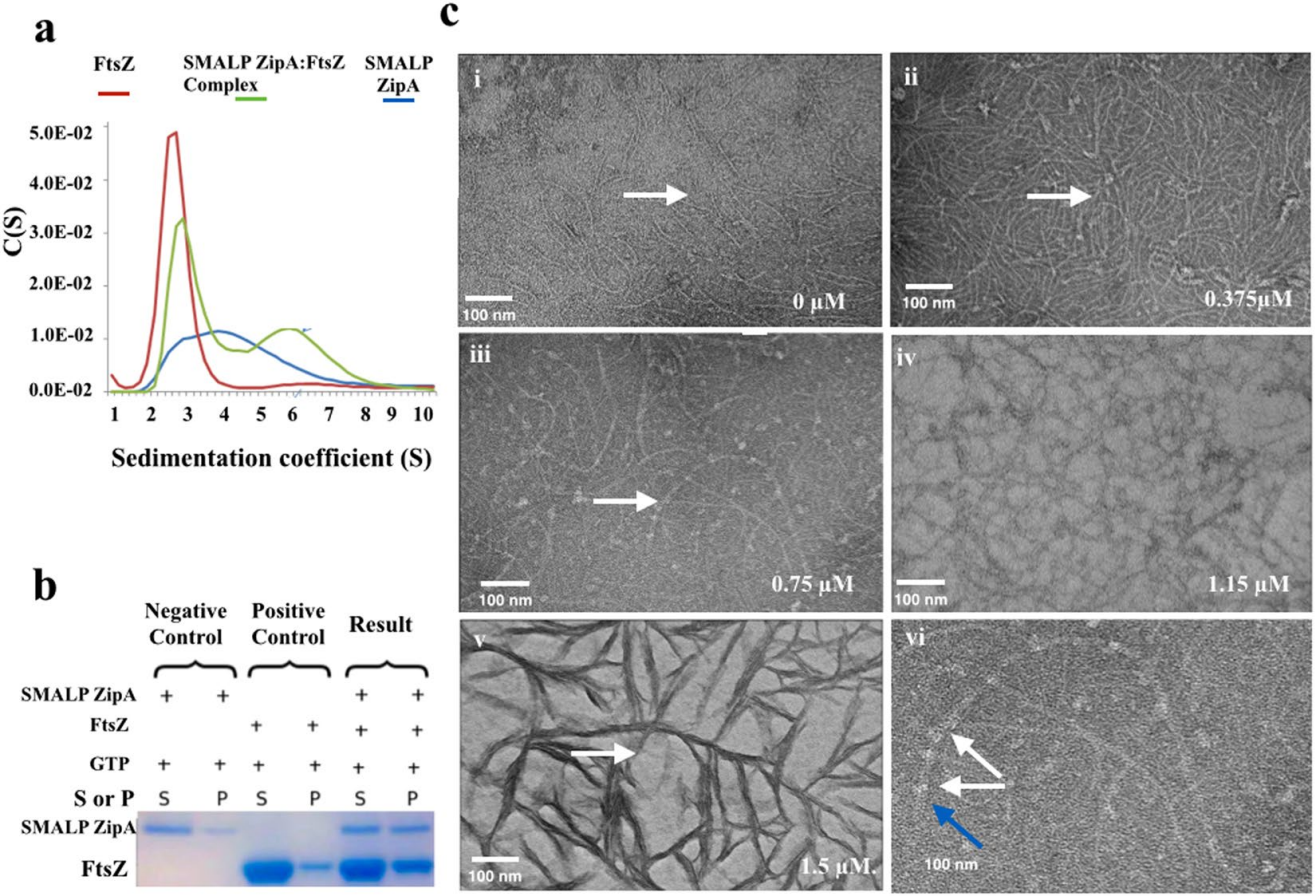
Interaction of SMALP-ZipA with FtsZ as monomers and polymers. (**a**) SvAUC analysis of SMALP-ZipA (blue), FtsZ (red) and combination of FtsZ and ZipA in a 1:1 ratio (green). (**b**) SDS-PAGE Coomassie blue-stained gel for the Supernatant (S) and pellet (P) of FtsZ (11 μM) and SMALP-ZipA (3.3 μM) (Lanes 5 and 6), with negative control, ZipA (3.3 μM Lanes 1 and 2) and positive control, FtsZ (11 μM Lanes 3 and 4) all in polymerisation buffer (50 mM MES pH 6.5, 50 mM KCl, 2.5 mM MgCl_2_ and 2 mM GTP. (**c**) TEM negative stain micrographs show the effect of increasing amounts of SMALP-ZipA on FtsZ (5.5 µM) in polymerisation buffer. SMALP-ZipA concentrations (i) 0 µM, (ii) 0.375 µM, (iii) 0.75 µM, (iv) 1.15 µM, (v)1.5, (vi) expanded 0.75 µM. White arrows point to the fibre and blue arrow indicates SMALP-ZipA.

Previous studies of full-length and modified ZipA by De Boer *et al*.^32^ have shown that it has a number of observable functions. Firstly ZipA binds to FtsZ when present as a monomer *via* the FtsZ CCTP^4^. Secondly, ZipA is able to induce FtsZ polymerized fibres to associate laterally to form bundles. Therefore, to establish the functionality of SMALP-ZipA we investigated the capacity of SMALP-ZipA to bind to monomeric FtsZ. Association of a stable complex was confirmed by SvAUC (Fig. 3a). Here SMALP-ZipA and FtsZ alone can be seen as single peaks indicating their mono-dispersity. When SMALP-ZipA was added to FtsZ at equal concentrations it resulted in the appearance of a higher molecular weight peak that did not correspond to those expected for either SMALP-ZipA monomer or FtsZ alone. This new peak corresponds to a sedimentation coefficient of 6.2 S consistent with a molecular weight of 100 kDa, with a frictional ratio of 1.2. Thus we conclude that SMALP-ZipA binds to monomeric FtsZ. The presence of a SMALP-ZipA:FtsZ complex was further confirmed using a sedimentation assay. SMALP-ZipA and FtsZ were mixed together at equal concentrations with and 2 mM GTP was added to the mixture to induce the polymerisation of FtsZ allowing it to be sedimented. The resulting pellets and supernatants were analysed by SDS-PAGE (Fig. 3b, full length gels are presented in Supplementary Fig. 1). These data show that SMALP-ZipA co-sediments with polymerised FtsZ clearly demonstrating that SMALP-ZipA also binds to polymeric FtsZ.

A key function of ZipA is to induce the organization of FtsZ fibres into larger laterally associated structures alongside a kinetic stabilization of the fibre^44^. To examine whether SMALP-ZipA could participate in similar interactions, samples containing varying ratios of SMALP-ZipA and FtsZ with GTP were imaged by negative stain TEM. The Fig. 3c images show that increasing the ratio of SMALP-ZipA to FtsZ in polymerisation buffer increases the thickness of the FtsZ fibres with progressively increasing bundling and cabling as the concentration increases. Without SMALP-ZipA fibres form as single polymers (Fig. 3c(i)). At 15:1 FtsZ:SMALP-ZipA more single-stranded organised fibres are seen, with some slight decoration by SMALP-ZipA observed (Fig. 3c(ii)). At 7:1 FtsZ:SMALP-ZipA double stranded fibres can clearly be observed which are decorated at regular intervals with SMALP-ZipA (Fig. 3c(iii)). The magnified pictures make the SMALP-ZipA decorated double cables more obvious (Fig. 3c(vi)). The lower binding ratio images (Fig. 3c(iv and v)) are dominated by thicker strands of FtsZ with SMALP-ZipA obscured.

### Structural investigation of SMALP-ZipA

#### Electron Microscopy

**Figure 4:**
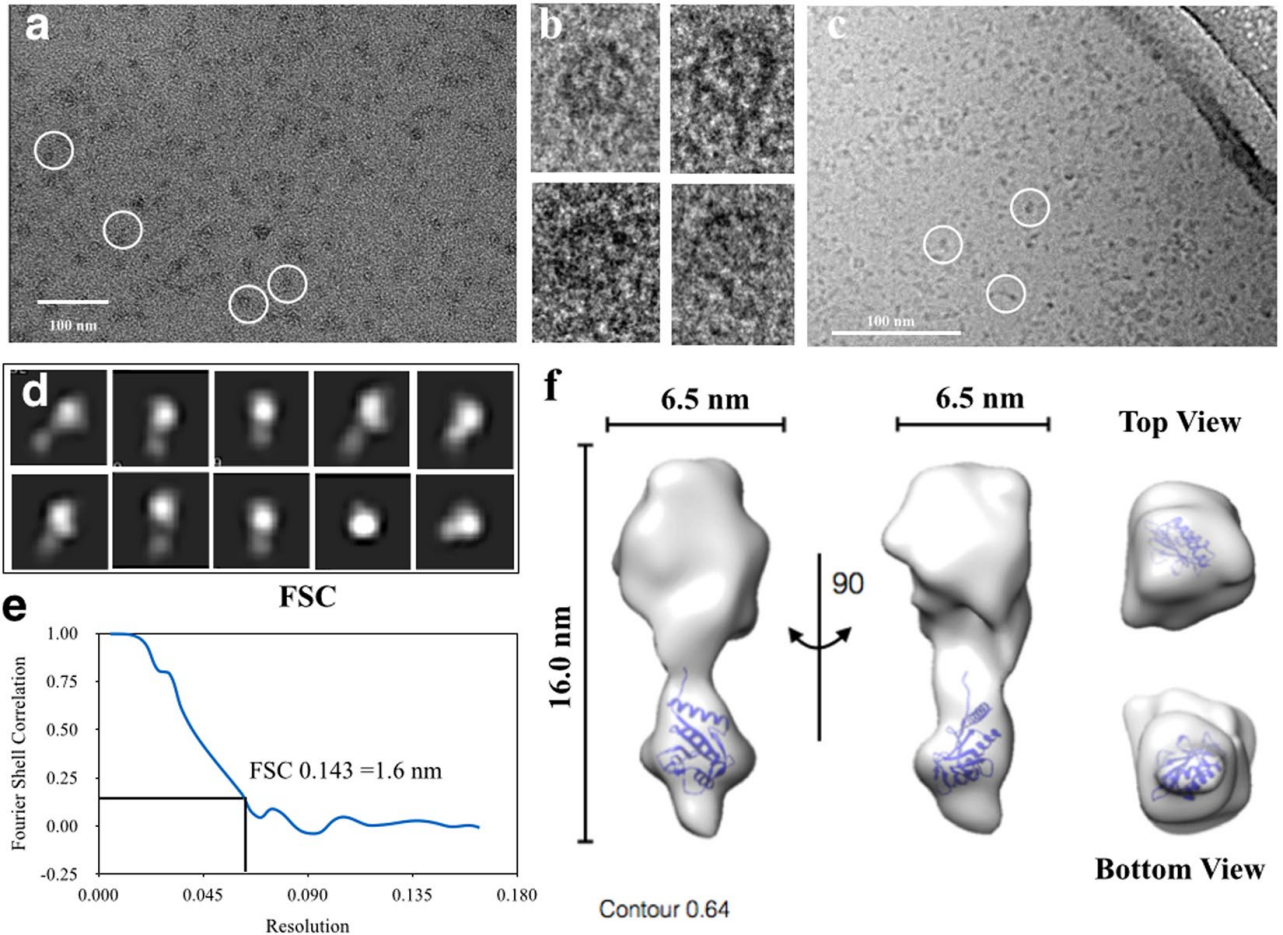
TEM of SMALP-ZipA. (**a**) Negative stain micrograph (2% Uranyl acetate) of SMALP-ZipA after SEC, selection of individual particles are illustrated with white circles, (**b**) Individual particles from the micrographs are illustrated to show SMALP-ZipA particles. (**c**) Cryo-EM image of SMALP-ZipA, the edge of the hole in the carbon support film of the EM grid is visible at the top right of the image. The particles highlighted by white circles are 10–16 nm in diameter and are relatively monodispersed. (**d**) Classification of the particles reveals elongated shape of approximately 6.5 nm in width and 16 nm in length. The box size is 19.52 nm. (**e**) further refinement of the structure against all the raw particles. Fourier shell correlation (FSC) for the refined structure using the gold standard 0.143 gives a resolution of 1.6 nm. (**f**) The 3D reconstruction of SMALP-ZipA using EMAN2 is displayed with the X-ray crystal structure of ZipA GD (PDB 1F47 with FtsZ 17aa C-terminal peptide) docked within the area protruding from the nanodisc (blue ribbon) showing very good fit. Orthogonal views are shown, giving a length of 16.nm, and width of 6.5 nm.

Negative stain TEM was used to investigate the size and shape of the SMALP-ZipA, after SEC (Fig. 4a). This revealed a mono-dispersed population of particles with a homogeneous size distribution. The SMALPs have a size of ~10 nm consistent with previous observations of MP’s in SMALPs^23,45^ and comparable to lipid-only SMALPs^46,47^. Views of typical particles from the micrographs can be seen in Fig. 4b. Although the protein is small (~ 40 kDa), and outside the normal range of proteins that are amenable to this type of approach, the overall SMALP-ZipA particle is large enough to be observed in Cryo-EM (Fig. 4c), with the lipid and SMA providing added mass which enabled determination of a low resolution 3D map. Cryo-EM images of 17000 particles were collected and a structure reconstructed from the data (Fig. 4d) with a resolution estimate of 1.6 nm using a Fourier shell correlation (FSC) gold standard of 0.143 as a criterion (Fig. 4e).

An initial examination of the structure using the Chimera visualisation tool^48^, shows a bi-lobular particle with larger disc-shaped density a smaller protrusion extending along the plane of the larger disc (Fig. 4f). The upper nanodisc has a diameter 6.5 nm and thickness that is slightly smaller than those observed for SMALPs alone, and a length of 16 nm. It therefore seems logical to conclude that the protrusion under the rim of the disc is the ZipA globular domain. To test this conclusion, the X-ray crystal structure of ZipA GD was fitted into the Cryo-EM density. This showed that the ZipA GD is of a size compatible with the small protrusion on the Cryo-EM structure. Views from the top looking down, and the bottom looking up also indicate that this is a good fit (Fig. 4f).

#### Small Angle X-ray Scattering

**Figure 5:**
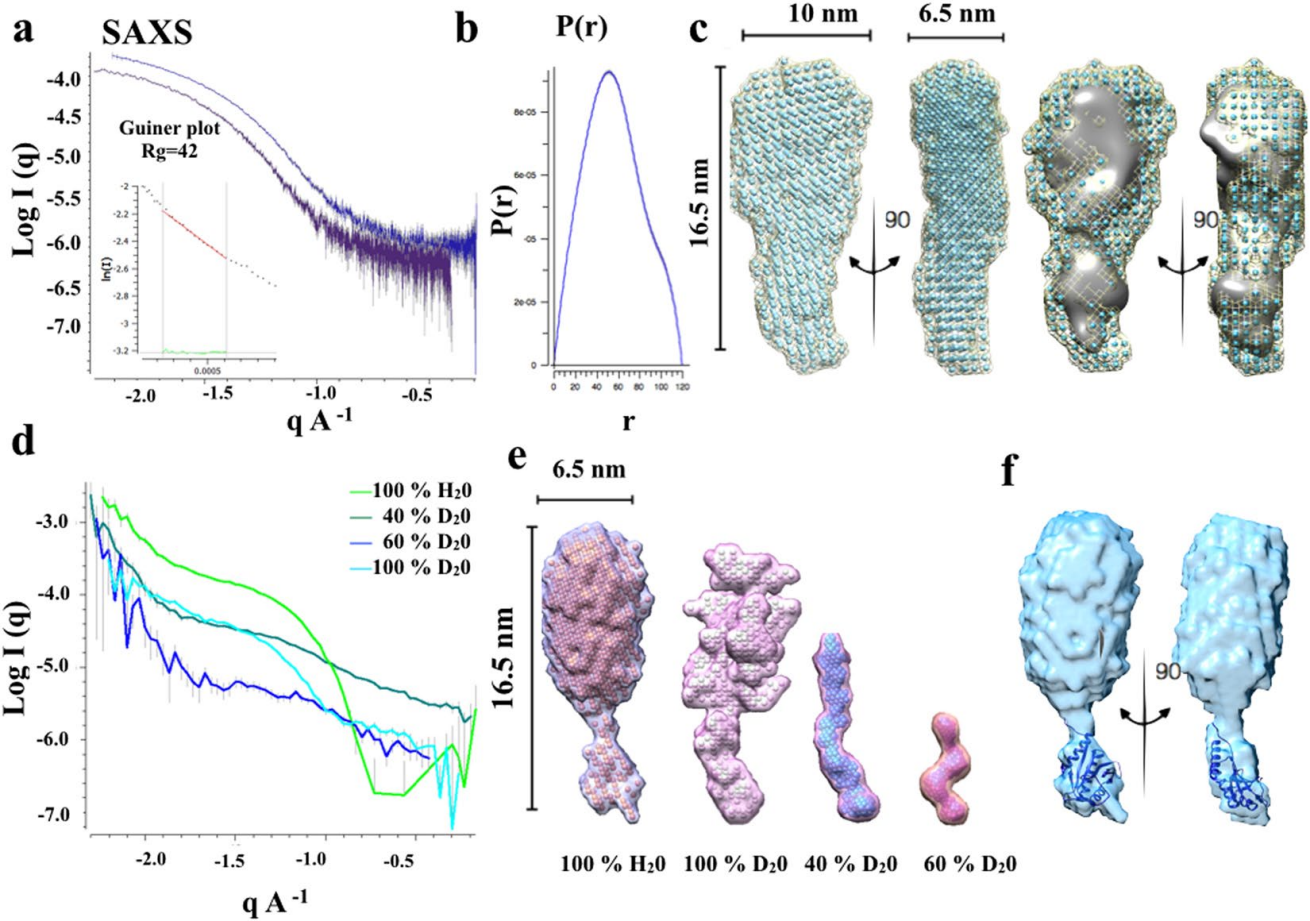
SAXS and SANS2D analysis of SMALP-ZipA. (**a**) Experimental SAXS curves of SMALP-ZipA. A straight-line in the Guinier plot is indicative of Guinier scaling with R_g_ of 4.2 nm. (**b**) Pair distribution function P(r) of SMALP-Zip shows a D_max_ of 12 nm. (**c**) Dummy atom model of the SAXS reconstructed envelop from DAMMIF, showing orthologous views. The dimensions show a length og 16.5 nm, width 10 nm, and 6.5 nm for side-on or face-on view respectively. Front and side views are of docking SMALP-ZipA Cryo-EM structure file (.pdb file) into the DAMMIF envelope are displayed. (**d**) SANS2D data collected of SMALP-ZipA in four different contrasts of H_2_O/D_2_O, shows different features in each data point (**e**) DAMMIF models of SANS2D data at four different contrasts. (**f**) Orthogonal views of surface contour of SMALP-ZipA DAMMIF SANS2D model with the X-ray crystal structure of the GD of ZipA (1F47).

To further confirm the structure deduced by the Cryo-EM 3D reconstruction model and literature crystal data, we carried out SAXS experiments on a solution containing SMALP-ZipA. Figure 5a represents scattering curves for SMALP-ZipA. The scattering curves obtained from SAXS measurement shows that the data are good as judged by the Guinier plot which is indicative of Guinier scaling (Fig. 5a). The P(r) function for the sample is also displayed (Fig. 5b). The maximum dimensions, D_Max_, for the sample is 12 nm while the radius of gyration, R_g_, is 4.2 nm. The data show that the sample is mono-disperse while the shape of the P(r) plot demonstrates that there is a small amount of flexibility in SMALP-ZipA. This fits with a model where the ZipA GD emerges from the SMALP disc and a small amount of flexibility is seen. The data were used to produce a dummy atom model using routines in PRIMUS^49^ which uses DAMMIF^50^, and DAMAVER^51^. To compare the Cryo-EM model with that calculated from the SAXS data, these data sets were contoured at 1 nm in Chimera (Fig. 5c) and fitted together. This overlay showed that the two structures fit well with respect to one another clearly indicating that the Cryo-EM structure is a good representation of the structure of SMALP-ZipA in solution.

#### Small Angle Neutron Scattering

The relatively low resolution of the Cryo-EM and SAXS data, mean that the assignment of regions that represent the SMALP and ZipA is challenging. As shown in the preceding sections the geometric features of the structure do fit with the known structures of the SMALP and ZipA. However, to provide more confidence that this assignment is correct, neutron scattering experiments were carried out. This included data collection using a number of different D_2_O contrasts that allows various elements of the structure to be contrast matched from the overall structure. This allows the density relating to the lipid in the SMALP, or the peptide that makes up ZipA, to be positively identified within the dummy atom model. Figure 5d shows SANS2D data for SMALP-ZipA collected at four different D_2_O contrasts. After buffer subtraction using MantidPlot, the data were analysed in a similar manner to the SAXS data. The scattering curve and the dummy atom model obtained for the 100 mol % H_2_O sample showed similarity to the molecular envelopes already determined using Cryo-EM and SAXS and are displayed in Fig. 5e. This further confirms that the structure represents that of SMALP-ZipA in solution. It is possible to fit the ZipA GD X-ray crystal structure into the protruding density below the nanodisc in all orientations (Fig. 5f). Analysis of the structures produced at different contrasts showed that, as the concentration of D_2_O increases towards 40 mol %, the density of the SMA and lipid gradually disappears from the dummy atom model. At 40 mol % the structure shows both the transmembrane region and ZipA GD with little or no density from the SMA and lipid. At this D_2_O concentration the scattering from lipid component of the nanodiscs is close to zero, this is the result that might be expected. Therefore, the data confirm our assignment of Cryo-EM density to protein and SMALP-ZipA.

### Structural determination of the SMALP-ZipA:FtsZ complex

**Figure 6:**
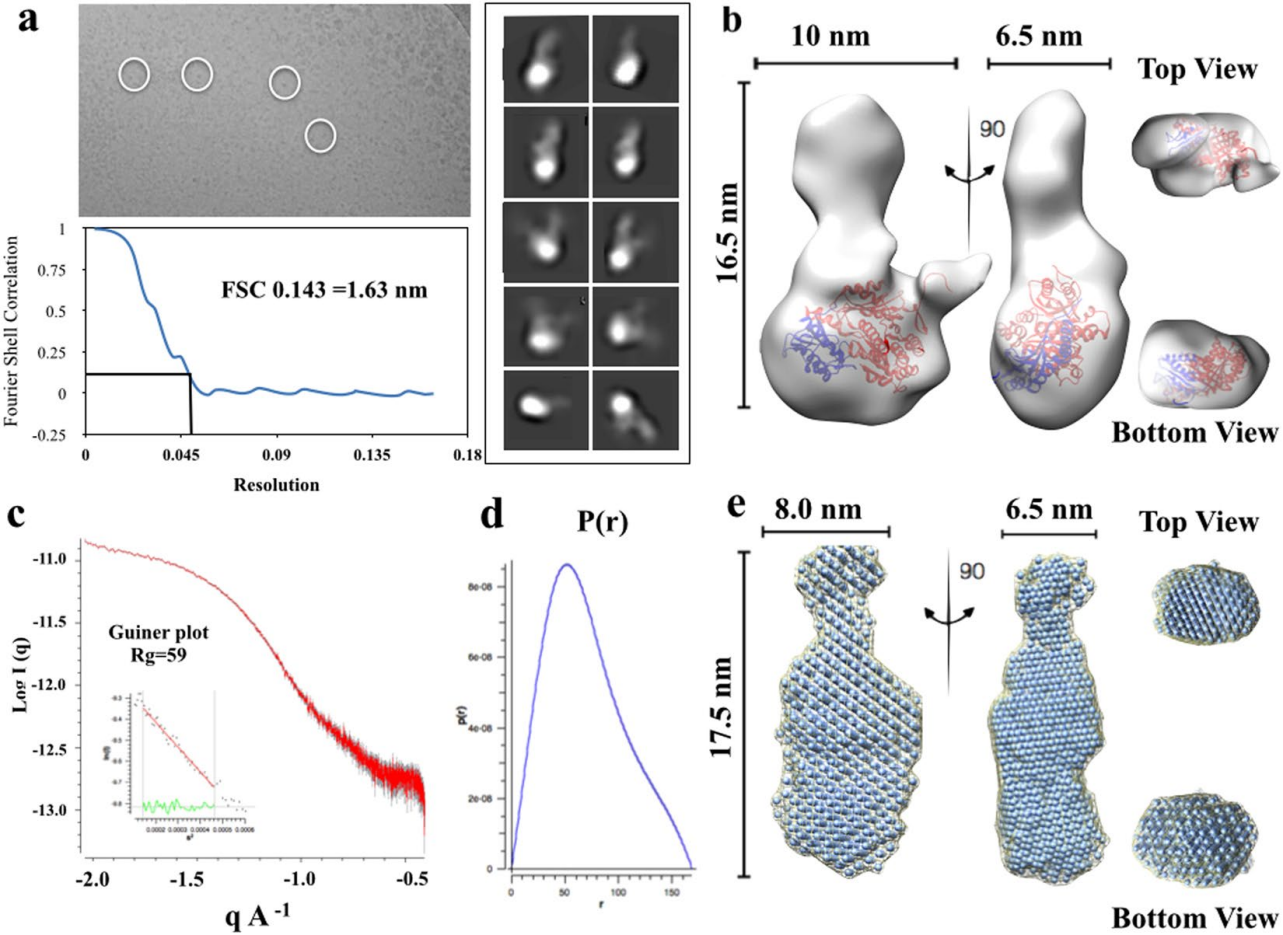
TEM and SAXS analysis of SMALP-ZipA:FtsZ complex. (**a**) Cryo-EM image of SMALP-ZipA:FtsZ, the edge of the hole in the carbon support film of the Cryo-EM grid is visible at the top right of the image, Class averages and Fourier Shell Correlation (FSC) at gold standard 0.143 (**b**) 3D reconstruction using EMAN2 of SMALP-ZipA:FtsZ, X-ray crystal structure of the GD of ZipA (PDB 1F47 with FZB PEP and 1FSZ blue and red ribbon) docked within the area protruding from the nano-disc (uppermost). Estimated resolution 1.6 nm resolution. (**c**) Scattering curve of SMALP-ZipA:FtsZ showing Guiner plot (**d**) Pair distribution function P(r) of SMALP-ZipA:FtsZ shows a D_max_ of 15.8 nm. (**e**) Dummy atom model of the SAXS reconstructed envelop from DAMMIF, orthologous views are shown.

Cryo-EM structural determination of the SMALP-ZipA:FtsZ complex was carried out on a sample of the complex purified using SEC.Twenty micrographs were collected and the data was analysed using EMAN2 software (Fig. 6a). Classification of the particles reveals elongated particles of approximately 10 nm or 6.5 nm in width, and 16.5 nm in length. 32 class averages were used to further refine the structure against all the raw particles.

The 3D reconstruction produced an envelope with increased volume density compared to that for SMALP-ZipA (Fig. 6b). This suggests that the particle contains not only SMALP-ZipA but also FtsZ. The structure is bi-lobular, in a similar way as SMALP-ZipA, however, one of the lobes is significantly larger than either of those observed in SMALP-ZipA. Current structural information on the structure of the ZipA:FtsZ complex comes from an X-ray crystallographic structure of the ZipA GD from *E*. *coli* bound to a small peptide with a sequence corresponding to the C-terminal domain of FtsZ^4^. Sequence similarities between *E*. *coli* FtsZ and other structurally determined FtsZ allowed the production of a putative model of the complex. Unfortunately, the C-terminal domain of FtsZ responsible for ZipA binding is not present in any existing x-ray crystal structures. This means that any model of a complex has to be rudimentary. In our case we produced a model by placing the *E*. *coli* ZipA structure close to *Methanocaldococcus jannaschii* FtsZ with the truncated C-terminus of FtsZ close to the known FtsZ binding region on ZipA. A model was generated of the complex using the x-ray crystal structure of the Globular Domain (GD) of ZipA (from pdb file 1F47)^4^ and FtsZ (pdb file 1FSZ)^52^ which contains the FtsZ-binding domain peptide (blue and red) docked within the area protruding from the nano-disc (uppermost). The refined structure was estimated to be at approximately 1.6 nm resolution. Given this complex, analysis of Cryo-EM envelope produced for SMALP-ZipA alone showed that it was not large enough to fit the complex. However, if the complex is fitted to the Cryo-EM envelope calculated for SMALP-ZipA:FtsZ a good fit is achieved with the ZipA:FtsZ complex placed in the larger of the two lobes. SAXS data was also collected for this complex (Fig. 6c), the dimensions of which agree with those obtained for the Cryo-EM reconstruction. The scattering curves obtained from SAXS measurement shows that the data are good as judged by the Guinier plot which is indicative of Guinier scaling (Fig. 6c) and the P(r) function for the sample is also displayed (Fig. 6d). Experimental SAXS curves of SMALP-ZipA:FtsZ gives a straight-line Guinier plot which is indicative of Guinier scaling gives a R_g_ of 5.9 nm.The D_Max_ for the sample is 15.8 nm while the radius of gyration, R_g_ is 5.9 nm. The data show that the sample is mono-disperse while the shape of the P(r) plot demonstrates that there is a small amount of flexibility in SMALP-ZipA:FtsZ. A dummy atom model produced using DAMMIF in a similar way to SMALP-ZipA shows that the structure produced using SAXS agrees well with that from Cryo-EM d with the length being 17.5 nm, and the widths 8 nm and 6.5 nm (side on or face on views). Front and side views of docking the SMALP-ZipA Cryo-EM structure file (.pdb file) into the DAMMIF envelope are displayed (Fig. 6e).

#### Fluctuating Finite Element Analysis of the Molecular Dynamics of SMALP-ZipA and SMALP-ZipA:FtsZ

**Figure 7:**
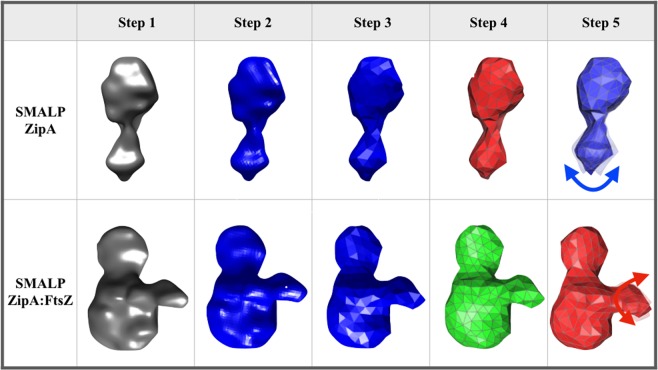
The steps taken to build the FFEA model for SMALP-ZipA and SMALP-ZipA:FtsZ from Cryo-EM density maps. Step1)The electron density profile is determined and formatted as a Cryo-EM.hdf file, Step 2) a triangulated surface is generated from the.hdf file using an appropriate contour level, Step 3) the triangulated surface is passed through an edge collapse algorithm to reduce the resolution and complexity, Step 4) the volume bounded by the (closed) surface is fully populated with tetrahedra using NETGEN, a 3D mesh generator^81^. The final connected tetrahedral mesh allows the SMALP structures to be analysed using FFEA continuum simulations. Step 5) The range of movement for SMALP-ZipA shows that the major mode of flexibility involves a bending motion between the ZipA GD and the PQ domain, (blue arrow). The range of movement for SMALP-ZipA:FtsZ complex instead involves a correlated bending motion between the FtsZ protrusion and the PQ domain. The radius of movement is indicated by the red arrow, and is calculated from FFEA to be in the region of 1.18 nm.

One of the questions that remains regarding the complex between FtsZ, ZipA and the membrane is the flexibility of the overall complex. Previous work has proposed that the unstructured region of ZipA provides a highly flexible linker that allows the globular domain of ZipA considerable steric freedom. Using the structural data obtained *via* Cryo-EM, we have been able to use Fluctuating Finite Element Analysis (FFEA)^53–57^ to calculate the conformational space explored by SMALP-ZipA and SMALP-ZipA:FtsZ. FFEA is a new modelling technique which treats globular bio-molecules as viscoelastic, continuum mechanical materials that change shape due to thermal fluctuations^58^. FFEA models resemble Gaussian/Anisotropic Network Models^59^, but since there is no need for “particles” in the continuum limit, they avoid the need to arbitrarily assign dummy atoms within a 3D volume envelope when atomistic information is unavailable. The Cryo-EM density maps for SMALP-ZipA and SMALP-ZipA:FtsZ were sufficient to construct a 3D tetrahedral finite element mesh for each complex (Fig. 7).

To generate dynamics within FFEA, the volumetric meshes require material parameters to be defined; most important is the Young’s modulus, since this determines the deformability of the protein. We chose our Young’s modulus, *E*, based on the reported values for the Young’s modulus of globular proteins as measured using nano-indentation experiments^60^, which all lie above 0.5 GPa, with significantly larger values having been reported for filamentous proteins, such as collagen and amyloid fibrils. Theoretical work by Kim *et al*.^61^, who performed automated normal mode analysis on finite element meshes formed from structures of well folded macromolecular protein complexes deposited in the Electron Microscopy Data Bank (EMDB)^62^ determined that a Young’s modulus of between 2 GPa and 5 GPa, together with a Poisson’s ratio of 0.3, is appropriate for modelling biological complexes.

To determine the dynamical behaviour of the SMALP-ZipA and SMALP-ZipA:FtsZ complexes, we performed three 1 µs FFEA simulations of each molecule, each homogeneously parameterised using three different values of the Young’s modulus: 0.5 GPa, 1 GPa, and 2 GPa. Following these simulations, we performed principal component analysis (PCA)^63^ on the resultant sets of 5000 trajectory frames from each simulation. This generated a set of eigenvectors, corresponding to the elastic normal mode motions, and eigenvalues, corresponding to their respective effective flexibilities. For both the SMALP-ZipA and SMALP-ZipA:FtsZ models, each of the three different parameterizations yielded effectively identical sets of eigenvectors. This is the expected behaviour of PCA analysis in this case, as we kept the distribution of material parameters constant throughout each molecule.

**Figure 8:**
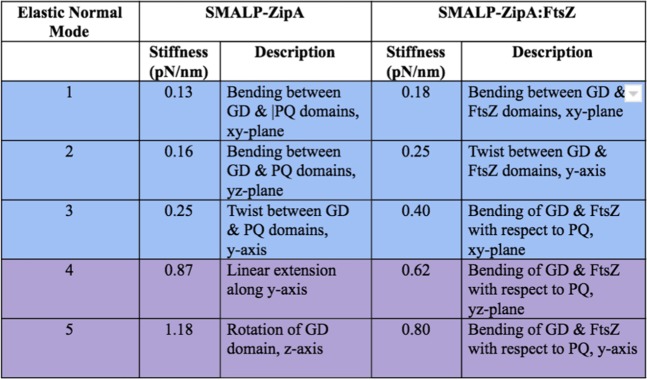
FFEA analysis showing the effective stiffnesses of each elastic normal mode calculated via PCA using a Young’s Modulus of 1 GPa.

Direct comparison of the eigenvalues of these modes, is shown in Fig. 8. FFEA analysis shows the effective stiffnesses of each elastic normal mode calculated via PCA using a Young’s Modulus of 1 GPa. The stiffnesses of the protein density of SMALP-ZipA and SMALP-ZipA:FtsZ is calculated from the raw variance values using the equipartition theorem at a temperature of *298 K*. A description of the motion corresponding to each eigenvector for the five most flexible modes is provided alongside the calculated stiffness (in pN per nm). When comparing mode 1 for SMALP-ZipA with mode 3 for SMALP-ZipA:FtsZ, which are associated with a bending between the GD and PQ domains, SMALP-ZipA:FtsZ is less flexible by ~33% shows that the addition of the FtsZ complex to the model increases the stiffness of this major mode by a factor of approximately 3. The five lowest frequency normal modes for both SMALP-ZipA(SMALP-ZipA_mode1-5)and SMALP-ZipA:FtsZ (SMALP-ZipA_FtsZ_mode1-5) are supplied (Supplementary movies).

We see that the overall motion displayed in SMALP-ZipA is perturbed when bound to FtsZ, such that motion becomes centred on the protrusion on the side of nanodisc. This suggests that the binding of ZipA to FtsZ affects ZipA by altering its dynamics in the membrane. We also note that the conformational change associated with the major mode of SMALP-ZipA is approximately equivalent to the third most dominant mode of the SMALP-ZipA:FtsZ complex.

The PCA major mode of flexibility (bending) and the radius of movement of the PQ and globular domain of SMALP-ZipA, indicated by the blue arrow, allows for only limited movement when in the SMALP nanodisc.

## Discussion

Cell division requires remodelling of the membrane in order for the cell to form a partition and divide into two daughter cells. We are now aware of some exceptionally intricate protein machinery to achieve these complex morphological changes in different organisms. With respect to bacteria, researchers studying this process over the last 20 years have carried out a large number of experiments to identify the members of this protein machinery and map their interactions. Many proteins are membrane associated, as would be expected given the process, making the study of the structure and function of these proteins using traditional biochemical methods exceptionally challenging. Central to this challenge has been the difficulties that plague all MP biology, that of extraction and purification.

In this study we have employed a new extraction method using SMALPs, which allows preservation of the lipid-protein complex to make functionally intact lipid-protein complexes. In this case, it allows visualisation of the structure of ZipA in the context of the membrane. The method is based on the use of the amphipathic polymer SMA. This method has preserved ZipA in its native state, and we confirm using CD, AUC and TEM, that the SMALP-ZipA is folded and monomeric. SMALP-ZipA, retains the activity expected for the protein and is able to bind to FtsZ monomers and polymers, showing that at a range of increasing concentrations it can induce and increase FtsZ fibre bundling.

The major aim of this work was to develop an approach to use the SMALP technology to gain information on the structure of ZipA and ZipA:FtsZ in its lipid environment. Structural information is now essential to provide deeper understanding of the division process, giving a detailed understanding of how changes in membrane morphology occur at the molecular level. Our previous studies of SMALP-protein complexes using TEM have shown that it is possible to obtain medium resolution data on the structure of proteins in these complexes^25,64^. However, in each of these cases the proteins were significantly larger than ZipA. In the context of Cryo-EM structural analysis, the size of ZipA (36.4 kDa) is at the lower end of what has been deemed possible^65^. Remarkably, our Cryo-EM data show that in fact the ZipA is visible within the SMALP structure which can be determined using 3D single particle analysis. This is, in part, due to the added mass that comes from the SMALP itself which contains both polymer and lipids. To confirm that the Cryo-EM structure is representative of the solution phase, we also determined structures using SANS2D and SAXS. This work demonstrates that these biophysical techniques are also able to give structural information from SMALPs although they are composed of three different densities (SMA, lipid and protein). Our results indicate that SMALP-ZipA is an asymmetric bi-lobular structure with the larger lobe structure is composed of lipid and SMA. The solvent contrast matching SANS2D experiments confirmed this interpretation showing that the larger lobe diminished in intensity under conditions that reduced the contrast of lipid and polymer.

Comparisons of the fundamental dimensions of the particles produced by SAXS, SANS2D and EM for SMALP-ZipA and SMALP-ZipA:FtsZ show the same overall dimension for the density related to the SMALP itself (SMALP-ZipA; EM: 6.5 × 6.5 nm; SAXS:10 × 6.5 nm; SANS2D: 6.5 × 6.5 nm. SMALP-ZipA:FtsZ; SAXS 6.5 × 8 nm.) These data also show that the size of the SMALP changes very little when FtsZ binds to the SMALP:ZipA. This is not unexpected given that the there are thought to be no interactions between the TM domain of the ZipA and FtsZ as it is buried in the membrane supported by the SMALP. An examination of the overall length of the particle also shows that this remains relatively unchanged upon binding of FtsZ to ZipA. This indicates two things, one that the binding site on ZipA for FtsZ does not occur on the apical surface of ZipA (furthest from the membrane) as this would result in a significant increase in length of the particle. In addition, it indicates that the PQ domain of ZipA does not substantially extend, something which would again lead to an increase in the length of the particle.

Molecular docking demonstrating that the X-ray crystal structure of the ZipA GD, fits well into the smaller lobe in all dimensions of the 3D structures in all three models, determined using orthogonal structural techniques. This suggests that this small lobe is the ZipA GD with the rest of the protein being internal to the SMALP. Analysis of the final structure shows that the ZipA GD is separated from the lipid and SMA by a thinner region of density, likely to be the extended domain PQ, which forms a flexible tether between the ZipA GD and the 1 TMH fixed into the membrane.

The SMALP-ZipA:FtsZ complex shows a particle that is significantly larger than SMALP-ZipA alone. This structure is also bi-lobular with one lobe of similar size to that attributed to the lipid and SMA in the SMALP-ZipA structure and a second lobe that is larger. The larger lobe is consistent with models fitting a ZipA GD:FtsZ complex into this density. A static analysis of the structure shows that the area of the particle that correlates with unstructured region of ZipA remains relatively unchanged indicating that there is no significant conformational change in the presence of bound FtsZ.

Using FFEA simulations in conjunction with material parameters appropriate for folded proteins to examine the dynamic modes of both SMALP-ZipA and the SMALP-ZipA:FtsZ complex, we find that although there is flexibility in this unstructured region for SMALP-ZipA, the range of motion is restricted. Nevertheless, it could still provide enough flexibility to enhance the interactions between ZipA and the FtsZ fibre near the membrane. Thus, it seems that the unstructured region allows ZipA to engage in a limited amount of entropic seeking in this state. Interestingly, the binding of FtsZ effectively suppresses the bending mode of SMALP-ZipA, locking the complex into a more rigid conformation.

The negative stain study presented showing the interaction between SMALP-ZipA and FtsZ polymers are in accord with the known simultaneous localisation of ZipA and FtsZ to the Z-ring^26^ and show that despite its bulk, the SMA lipid particles do not disrupt the structure of the binding motifs of ZipA nor access to the FtsZ binding domains. In fact, it suggest that the simplistic cookie cutter visualisation of the SMALP is appropriate with the SMA wrapping around lipids and the buried portions of ZipA but leaving its hydrophilic parts exposed to enable its normal FtsZ binding activity with the bundled geometries avoiding the SMALP just as they accommodate a membrane surface. The fact that SMALP encapsulated ZipA binds to monomers indicates that the ZipA site does not span multiple FtsZ molecules, in contrast to ZapA which reduces the rate of hydrolysis facilitating longer polymers and enhanced cell division^66^ which is in accord with it having no effect on GTPase activity of FtsZ^67^ Based on these data we speculate that the ZipA is present in the membrane and attracts FtsZ monomers to bind. When an FtsZ molecule is incorporated into the ring, a ZipA is automatically included and ensures membrane binding.

**Figure 9:**
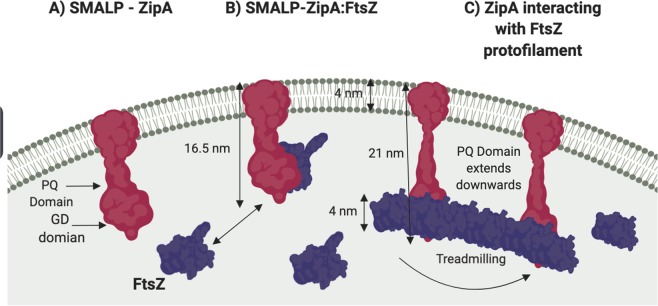
Schematic representation of the interaction between ZipA and FtsZ. (**A**) SMALP-ZipA is represented in the membrane (Pink). (**B**) ZipA:FtsZ complex, FtsZ (Purple) binds ZipA GD domain via the GD peptide to one side of ZipA. Tethered to the membrane, ZipA also holds FtsZ near the membrane. (**C**) During protofilament formation, ZipA PQ domain is able to extend (uncoil) into the cell, and tethers FtsZ protofilament whilst it undergoes extension and associated treadmilling. (Created with Biorender.com).

To relate experimental data obtained from analysis of SMALP-ZipA and SMALP-ZipA:FtsZ a schematic representation (Fig. 9) demonstrates how the nanodiscs containing ZipA (Fig. 9A) and ZipA:FtsZ (Fig. 9B) can be represented in relation to the *E*. *coli* inner membrane. SMALP-ZipA is represented in a lipid bilayer of 4.0 nm^68^, with a length of 16.5 nm taken from experimental results. The position of the PQ domain and the ZipA GD are indicated relative to the membrane. Density is observed under the membrane which accounts for amino acid sequence between the 1TMH, PQ domain and the GD domain

Our findings show that the PQ domain is compact and packed underneath the disc, therefore underneath the membrane. It remains compact when bound to FtsZ as illustrated. Measurements of FtsZ fibres suspended from lipid membranes in model systems, and others who have modelled ZipA and FtsZ on bilayers suggest that the formation of the protoring from the lower leaflet of the membrane gives a distance of polymerises FtsZ 16-17 nm i.e total of 21 nm^6,33,69–73^.

 Here we suggest therefore that ZipA, tethered in the membrane and undergoes a conformational change, extending the PQ domain by lengthening this disordered domain. This allows the FtsZ polymers to be supported below the membrane, with the associated treadmilling^9,74^ of the ftsZ subunits, continuing to form the FtsZ proto-ring (Fig. 9C).

Analysis of SMALP-ZipA and SMALP-ZipA:FtsZ nanodiscs positively confirm that it is possible to obtain low resolution structural data from small MP’s such as ZipA. In addition the data show that the integrity of the ZipA protein is maintained in solution; something that is not easily achieved with standard MP purification methods. The SAN2DS model shows that the protein component of the SMALP-ZipA can be clearly discerned. This differs from previous work using the MSP nanodisc system^38^ where the authors struggled to discriminate protein density from that of the nanodisc. All these data produce molecular envelopes that are consistent with the model described in Fig. 1B,C. The compact nature of the model also challenges the view that the PQ domain is an unstructured free ranging peptide, but instead under these conditions, and in the confirmation used in this study, the ZipA GD is held firmly underneath the lipid SMA mantle like a mushroom, as described by Lopez-Montero *et al*.^34^.

Taken together the use of this MP extraction method in the context of bacterial divisome proteins provides valuable new insights into how these proteins interact with one another and at the membrane interface. This study paves the way for high-resolution studies of the SMALP-ZipA:FtsZ membrane complex and provides a route to isolation and high resolution imaging of divisome protein complexes bound to native lipid membranes for the first time.

## Methods

### Materials and SMA preparation

The preparation of Styrene Maleic Acid was prepared as outlined in Lee *et al*.^39^. Briefly, starting with a styrene-maleic anhydride copolymer reagent with a ratio of styrene to maleic acid of 2:1 (Cray Valley). The anhydride was converted to the acid using a basic hydrolysis protocol. 25 g of Styrene Maleic Anhydride Co-polymer was dissolved in 1 M NaOH and heated while and refluxing the solution. After cooling at room temperature (20 °C), Styrene Maleic Anhydride Co-polymer was precipitated by reducing the pH to below 5 by the addition of concentrated HCl, the pH is monitored at this stage. The precipitate was washed three times with water followed by separation using centrifugation. At the end of the third wash the precipitate was resuspended in 0.6 M NaOH. The solution was precipitated and washed again, and finally resuspended in 0.6 M NaOH. The pH was adjusted to pH 8. The polymer was lyophilised using a freeze dryer. The desiccated SMA copolymer powder was stored at room temperature in a sealed vessel.

### Gene expression and protein purification of SMALP-ZipA

ZipA was extracted from genomic DNA from *E*. *coli* strain TOP10 (invitrogen) and used as the template for a polymerase chain reaction (PCR) to amplify the ZipA gene. The gene was subsequently ligated into Champion pET101 directional TOPO plasmid vector containing a T7 promoter, a 6× His tag at C-terminus and Ampicillin resistance. Membranes containing ZipA bearing a C-terminal histidine tag, were prepared as described^39^. For preparation of SMALPs, membranes (40 mg) were incubated with gentle shaking for 2 h at room temperature in 32 ml 50 mM Tris-HCl, pH 8.0, containing 500 mM NaCl, 10% (wt/vol) glycerol and 2.5% (wt/vol) SMA. The insoluble fraction was removed by centrifugation at 4 °C for 1 h at 100,000 g and then the supernatant was incubated overnight at 4 °C with 2 ml of NiNTA resin (Generon) with gentle agitation. The resin was subsequently washed with 10 column volumes (cv’s) of 50 mM Tris-HCl, pH 8.0, containing 500 mM NaCl and 10% (v/v) glycerol before elution of the SMALP-ZipA with the same buffer containing 500 mM imidazole. Fractions containing SMALP-ZipA, were identified by SDS-PAGE analysis, pooled and concentrated using vivaspin (30 kDa MWCO), to a final volume of 250 ul. SEC was performed on the SMALP-ZipA to obtain SMALP-ZipA monomers. The sample was loaded on to AKTA FPLC using 50 mM Tris, 150 mM NaCl pH 8.0 as the running buffer at a flow rate of 1 ml min^−1^ through a Superdex 200 Increase column 10/300 GL (G.E Healthcare). 0.5 ml fractions were collected over the peak. A fraction containing mono-dispersed single particles were identified, by SvAUC and used for structural studies.

### Gene expression and purification of FtsZ

FtsZ 10 ml LB starter culture containing 100 µg/ml ampicillin (amp) was inoculated with a fresh colony of transformed *E*. *coli* BL21(DE3) pLysS cells (Invitrogen) and grown overnight at 37 °C with shaking at 180 rpm. The starter culture was inoculated into 1 L LB supplemented with 0.2% (w/v) glucose, and 100 μg/ml, incubated at 37 °C with shaking at 180 rpm until the optical density at 600 nm (1 cm pathlength) reached 0.6, compared to an LB blank. Protein expression was induced with the addition isopropyl β-D-1-thiogalactopyranoside (IPTG) at a 0.5 mM, for 4 hours at 37 °C with shaking at 180 rpm. Cells were harvested by centrifugation at 12,000 × g for 15 minutes at 4 °C. Cell pellets were stored at −20 °C. Following the method of Mukherjee and Lutkenhaus^75^, cell pellets were thawed, resuspended in 20 ml FtsZ Buffer A (50 mM Tris-HCl, 50 mM KCl, 1 mM EDTA, 10% (v/v) glycerol, pH 7.9) and held on ice and lysed by sonication then centrifuged at 50,000 × g for 45 minutes at 4 °C. The Supernatant was collected and FtsZ was precipitated with 1.7 g ammonium sulphate per 10 ml supernatant to give a 30% saturated solution. The solution was stirred for 20 minutes at 4 °C and centrifuged at 20,000 × g for 10 minutes at 4 °C. The protein pellet was resuspended in 10 ml FtsZ Buffer A and dialysed against 3 × 1 L FtsZ Buffer A. A 20 ml HiPrep 16/10 DEAE FF column (GE Healthcare) was used for anion exchange chromatography, and the protein was eluted over a gradient of increasing KCl concentration from 100% FtsZ Buffer A to 100% FtsZ Buffer B (50 mM Tris-HCl, 500 mM KCl, 1 mM EDTA, 10% (v/v) glycerol, pH 7.9) with a flow rate of 2 ml/min. FtsZ eluted from the column at a KCl concentration of 200–250 mM. 1.5 ml fractions were collected and fractions containing FtsZ were pooled. The protein was dialysed against 3 × 1 L IMAC Buffer A (25 mM HEPES, 0.5 M NaCl, 50 mM imidazole, pH 8) and was concentrated using a VivaSpin 20 10,000 MWCO PES membrane sample concentrator with centrifugation at 3,200 g at 4 °C. A 5 ml HisTrap column (GE Healthcare) was used for IMAC. The column was washed with 50 ml dH_2_O, followed by 20 ml 50 mM EDTA, and 50 ml dH_2_O. The resin was re-charged with 10 mg/ml NiCl_2_ solution and washed with 50 ml dH_2_O. Finally, the primed column was equilibrated with 50 ml IMAC Buffer A. and the protein was loaded. The column was washed with 40 ml IMAC Buffer A then the protein was eluted over a gradient of increasing imidazole concentration from 100% IMAC Buffer A to 100% IMAC Buffer B (25 mM HEPES, 0.5 M NaCl, 500 mM imidazole, pH 8) over 50 ml with a flow rate of 2 ml/min. 1.5 ml fractions were collected and fractions containing FtsZ were pooled. The protein was dialysed against 3 × 1 L FtsZ Gel Filtration Buffer (50 mM Tris-HCl, 0.15 M NaCl, pH 8) and was concentrated using a VivaSpin 20 10 kDa MWCO PES membrane sample concentrator with centrifugation at 3,200 g at 4 °C. The FtsZ was further purified using a HiPrep 26/60 Sephacryl S-300 HR (GE Healthcare) column. The column was equilibrated with at least 2 cv’s of FtsZ Gel filtration Buffer using a flow rate of 2 ml min. After injection of the FtsZ solution, the flow rate was reduced to 1 ml min and 3 ml fractions were collected. Fractions containing FtsZ were pooled and dialysed against 3 × 1 L FtsZ Buffer A. FtsZ was concentrated to ~20 mg/ml using a VivaSpin 20 10 kDa MWCO PES membrane sample concentrator with centrifugation at 3,200 g at 4 °C, aliquoted and stored at −80 °C. Protein purity was assessed by SDS-PAGE, using 12% acrylamide resolving gels.

### Purification of SMALP-ZipA:FtsZ

Fractions containing SMALP-ZipA Monomers were mixed with purified FtsZ monomers. A 250 ul sample was loaded on to AKTA FPLC using 50 mM Tris, 150 mM NaCl pH 8.0 as the running buffer at a flow rate of 1 ml min^−1^ through a Superdex 200 Increase column 10/300 GL (G.E. Healthcare). 0.5 ml fractions were collected fractions were collected over the peak. SMALP-ZipA:FtsZ fractions were analysed by SDS-PAGE.

### Circular dichroism spectroscopy

CD spectra were measured in the far UV (190–260 nm) using a JASCO J-715 spectrophotometer and a 1 mm path length quartz cuvette containing 0.05 mg ml^–1^ of SMALP-ZipA. The data were collected with a data pitch of 0.5 nm and sixteen scans per measurement. A buffer spectrum collected with the same parameters was subtracted.

### Sedimentation velocity analytical ultracentrifugation

Twin channel AUC cells were prepared with 400 μl of SMALP-ZipA, SMALP-ZipA and FtsZ protein at a concentration between 0.1 and 0.5 mg ml^−1^ in one channel and 420 µl of relevant buffer blank in the second channel. The cells were loaded into a 50Ti rotor in a Beckman Coulter XL-I analytical ultracentrifuge (Beckman 693 Coulter). The centrifuge was operated at 129,000 *g* and at a temperature of 20 °C until the sample had fully sedimented. The protein within the cells was monitored by absorbance at 280 nm. Analyses of the data was undertaken the program SEDFIT^43^ using the c(S) and c(M) routines to provide estimations sedimentation coefficient and mass of the particle. Parameters for SMALP-ZipA protein partial specific volume, solvent density and viscosity were calculated using SEDNTERP.

### Negative stain transmission electron microscopy

Samples were visualized using negative stain transmission electron microscopy. Carbon films on 400 copper mesh grids (Agar Scientific) were glow-discharged for 20 seconds at 10 mA. 10 μL of the protein sample was applied to the grid. After 1 minute, excess liquid was blotted and the grid was stained with 10 μL 1% uranyl acetate solution. After 1 minute excess liquid was blotted. Grids were imaged using the JEOL 2011 electron microscope and software.

### **Cryo-electron microscopy and image reconstruction**

Electron microscopy grids (2 micron holes, Quantifoil) were prepared with SMALP-ZipA (0.3 mg/ml) using an FEI Vitrobot freezing device and by plunge freezing into liquid ethane. The grids were transferred into a Polara FEG transmission electron microscope maintaining the temperature at <100 K throughout. Areas with sufficiently thin ice were selected at low magnification and low dose, and micrographs were recorded on a 4k × 4k CCD device with 1 second exposure and low dose mode imaging conditions. Micrographs were analysed using the EMAN2 software package^76^, with >8000 particles selected from 20 images. Strong defocus was employed because of the relatively small mass of the particles (defocus range 3.5 to 5.9 micron). Correction for the contrast transfer function was carried out and then reference-free classification of the particles was performed with unique 32 classes identified. Preliminary 3D structures were generated from these classes using a common-lines algorithm and with no symmetry applied. The ten low resolution structures were similar and one was selected for refinement of the 3D structure using all the single particles. The resolution of the structure after 5 rounds of refinement was estimated by Fourier shell correlation between two structures generated from even- and odd numbered particles from the data set. The features observed in the final structure corresponded to the resolution estimate and with the strong suppression of higher resolution frequencies in the images as a result of the need for strong underfocus.

### Small angle x-ray scattering (SAXS)

SMALP-ZipA samples were prepared in 50 mM Tris pH 8.0, 150 mM NaCl, at a concentration of 1 mg.ml^−1^ The samples were measured using the using the Arinax BioSAXS sample handling robot on beamline B21 at Diamond Light Source, at the Rutherford Appleton Laboratory, Harwell, Oxfordshire, UK. The samples were run using the standard beamline configuration, at 12.4 keV, using the 1 mm thick quartz capillary on the sample-handling robot. Samples were pipetted into 96-well plates and run at 25 °C. Data was measured over a Q range of 0.008 and 0.4 Å-1, calibrated using silver behenate and reduced using the data reduction pipeline in DAWN. Measurements were taken as 60 frames of 1 second using a Pilatus 2 K detector. After checking to ensure no beam damage had occurred these 60 frames were averaged to provide the final measurement. An appropriate buffer background was subtracted from the sample scattering before data analysis.

Data was displayed analysed using ATSAS data analysis package, using PRIMUS to determine Guinier plot and P(R) function, and to determine R_g_ and D_max_ values for SMALP samples. DAMMIN^50^ and DAMMIF^50^ analysis was performed from within PRIMUS and best fit achieved from iterative processing to achieve Dummy atom models, models produced were displayed in Chimera^48^ by displaying data as spheres. The dummy atom model was then contoured at 1.5 nm in Chimera^48^ to provide a shell, and fitted into the Cryo-EM map^77^.

### Small angle neutron scattering (SANS2D)

SMALP-ZipA samples prepared in range of 50 mM Tris pH 8.0, 150 mM NaCl buffers proving range of contrasts of H_2_O and D_2_O to give a final concentration of 1 mg ml^−1^ The solutions were measured on the SANS2D instrument^78^ at the SFTC ISIS Spallation Neutron and Muon Source, at the Rutherford Appleton Laboratory, Harwell, Oxfordshire, UK. SANS2D is a time-of-flight instrument with two movable detectors. For this measurement, the rear detector was placed at a sample to detector distance of 4 m, giving a Q range of 0.007 to 0.32 Å^−1^. Samples were run in 1 mm thick, 1 cm wide quartz Hellma cells at 25 °C. Data was reduced using the standard routines within MANTID^79^. The data was normalised to the sample transmission, corrected for detector efficiencies and appropriate buffer backgrounds were subtracted before further analysis. These data were displayed and analysed using ATSAS data analysis package, using PRIMUS to determine Guinier plot and P(R) function, and to determine R_g_ and D_max_ values for SMALP samples.

### Simulations of SMALP-ZipA, and SMALP-ZipA:FtsZ Complex using FFEA

All simulations were performed using the FFEA simulation software^57^, based on FE meshes generated with NETGEN 6.0. We performed three simulations of both the SMALP-ZipA and SMALP-ZipA:FtsZ complex, each with Poisson ratio *ν* = 0.35 but with Young’s moduli of 0.5 GPa, 1 GPa and 2 GPa. This gives us a distribution of flexibilities for comparison at the upper limit of possible range for biomolecules. Earlier work by Oliver^56^ using FFEA to model axonemal dynein showed that protein dynamics are only weakly affected by the Poisson ratio, *ν*, for perturbations about a value *ν* = 0.35. We also define shear and bulk viscosities within the molecule, *µ*_*S*_ and *µ*_*B*_ respectively, as well as an external viscosity *µ*_*E*_. Each of the viscosities *µ*_*S*_ = *µ*_*B*_ = *µ*_*E*_ = 1 × 10^−3^ Pa.s, approximately equivalent to water at room temperature^80^. This parameterization yields models with realistic spatio-temporal properties for simulation. The numerical integration of the continuum equations of motion was performed using a timestep of 10 fs. We calculated that the longest relaxation timescale associated with the normal modes was around 0.73 ns. Therefore, we obtain more than 1000 independent instances of the slowest mode from each 1µs simulation for statistical analysis in post-processing. PCA analysis was performed on the simulations by first transforming the raw FFEA trajectory data into PDB format, where each FFEA ‘node’ (tetrahedral vertex) was represented by a dummy carbon atom. These PDB files were then used as input into the pyPcazip software package^63^, from which we were able to extract the set of eigenvalues, and visualisations of the associated eigenvectors, corresponding to the positional variance accessible by each molecular complex.

## Supplementary information

Supplimentary Figure 1

SMALP-ZipA Movie 1

SMALP-ZipA Movie 2

SMALP-ZipA Movie 3

SMALP-ZipA Movie 4

SMALP-ZipA Movie 5

SMALP-ZipA:FtsZ Movie 1

SMALP-ZipA:FtsZ Movie 2

SMALP-ZipA:FtsZ Movie 3

SMALP-ZipA:FtsZ Movie 4

SMALP-ZipA:FtsZ Movie 5
