# Supplementary material for: Nano-encapsulated Escherichia coli Divisome Anchor ZipA, and in Complex with FtsZ
Source: Sci Rep. 2019 Dec 10;9:18712. doi: 10.1038/s41598-019-54999-x (PMC6904479; doi:10.1038/s41598-019-54999-x)
Supplement: Supplementary file 1 — Supplimentary Figure 1 [file 41598_2019_54999_MOESM1_ESM.docx]

**Nano-encapsulated *Escherichia coli* Divisome Anchor ZipA, and in Complex with FtsZ**

Sarah Lee^a^, Richard Collins^b^_,_ Yu-pin Lin^a^_,_ Mohammed Jamshad^a^_,_ Claire Broughton^c^, Sarah Harris^d^ Benjamin Hanson^d^, Cecilia Tognoloni^e^, Rosemary Parslow^a^ , Ann Terry^f^, Alison Rodger^g^, Corinne J. Smith^c^, Karen J. Edler^e^, Robert Ford^b^ ,David I. Roper^c^, & Timothy R. Dafforn^a^

^a^ School of Biosciences, University of Birmingham, Edgbaston, Birmingham B15 2TT, UK.

^b^ Faculty of Life Sciences, A4032 Michael Smith Building, Oxford Road, Manchester, M13 9PT, UK.

^c^School of Life Sciences, University of Warwick, Gibbet Hill Road, Coventry, CV4 7AL, UK

^d^School of Physics and Astronomy and Astbury Centre for Structural and Molecular Biology, University of Leeds, UK

^e^Department of Chemistry, University of Bath, Claverton Down, Bath, BA2 7AY, UK.

^f^ISIS, Rutherford Appleton Laboratory, Harwell Oxford, Didcot,OX11 0QX, UK

^g^Department of Molecular Sciences,Faculty of Science and Engineering Macquarie University, Sydney Australia.

Corresponding Authors: Professor Timothy R Dafforn, School of Biosciences and Dr. Sarah Lee, School of Biosciences, University of Birmingham, Birmingham, Edgbaston, B15 2TT U.K. Email: [T.R.Dafforn@bham.ac.uk](mailto:T.R.Dafforn@bham.ac.uk), [S.lee.5@bham.ac.uk](mailto:S.lee.5@bham.ac.uk) Tel: (+44) (0)121 414 3506.

**Supplimentary** Figure 1. Interaction of SMALP-ZipA with FtsZ

ZipA + + + +

FtsZ + + + + +

GTP + + + + + +

S or P S P S P S P


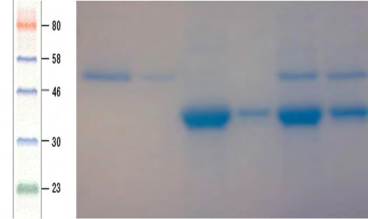


**ZipA**

**FtsZ**
